# Fitness cost from fluctuating ultraviolet radiation in *Daphnia magna*

**DOI:** 10.1098/rsbl.2021.0261

**Published:** 2021-08-04

**Authors:** Franca Stábile, Christer Brönmark, Lars-Anders Hansson, Marcus Lee

**Affiliations:** ^1^ Aquatic Ecology, Department of Biology, Lund University, Lund, Sweden; ^2^ Departamento de Ecología y Evolución, Facultad de Ciencias, Universidad de la República, Montevideo, Uruguay

**Keywords:** ultraviolet radiation, fluctuating environment, fitness, zooplankton, *Daphnia*, behavioural plasticity

## Abstract

Solar ultraviolet radiation (UVR) is an important environmental threat for organisms in aquatic systems, but its temporally variable nature makes the understanding of its effects ambiguous. The aim of our study was to assess potential fitness costs associated with fluctuating UVR in the aquatic zooplankter *Daphnia magna*. We investigated individual survival, reproduction and behaviour when exposed to different UVR treatments. Individuals exposed to fluctuating UVR, resembling natural variations in cloud cover, had the lowest fitness (measured as the number of offspring produced during their lifespan). By contrast, individuals exposed to the same, but constant UVR dose had similar fitness to control individuals (not exposed to UVR), but they showed a significant reduction in daily movement. The re-occurring threat response to the fluctuating UVR treatment thus had strong fitness costs for *D. magna*, and we found no evidence for plastic behavioural responses when continually being exposed to UVR, despite the regular, predictable exposure schedule. In a broader context, our results imply that depending on how variable a stressor is in nature, populations may respond with alternative strategies, a framework that could promote rapid population differentiation and local adaptation.

## 1. Introduction

In natural environments, organisms are exposed to various threats, and escaping from them generally implies a cost, in both energy and missed opportunities for feeding and reproduction. Depending on the nature, duration and predictability of the threat, different life strategies could arise [1]. In aquatic systems, solar ultraviolet radiation (UVR) is a temporally variable abiotic threat reported to have negative effects on a range of different aquatic organisms from different trophic levels, thereby structuring communities [2,3]. Increased mortality rates and reduced reproduction of several zooplankton species have been documented in response to UVR [4,5], as well as the induction of avoidance behaviours [6–8]. The zooplankton species *Daphnia magna*, in particular, has been repeatedly shown to exhibit strong negative phototaxis in response to UVR stress [8,9].

Most studies on effects of UVR have focused on effects from constant exposure as the treatment [8,10–13], despite the intensity of UVR in nature fluctuating strongly over short time scales with the position of the Sun and rapidly occurring variations in cloudiness. Several studies include these natural variations in UVR in their experimental design, but they do not explicitly address the costs of the fluctuating threat *per se* [4,14,15]. Yet, responding to these short-term fluctuations in UVR through avoidance behaviour likely implies a cost, in terms of both energy and missed opportunities for feeding and reproduction [8]. However, in a variable but predictable environment, phenotypically plastic responses could improve individual performance [16,17]. To our knowledge, no study has addressed how long-term, continuous fluctuations in UVR, mirroring the everyday environment in natural ecosystems, affect survival, reproduction and behaviour within a single generation. Therefore, the aim of this study was to assess the costs of fluctuating exposure of UVR in *Daphnia magna*, and we hypothesized that fluctuations in UVR would reduce the number of offspring and the survival of individuals owing to the allocation of energy to threat response movements. We also hypothesized that there may be behavioural plasticity in the individual responses, possibly accounting for part of the considerable variance observed in natural ecosystems.

## 2. Methods

**Figure 1.**
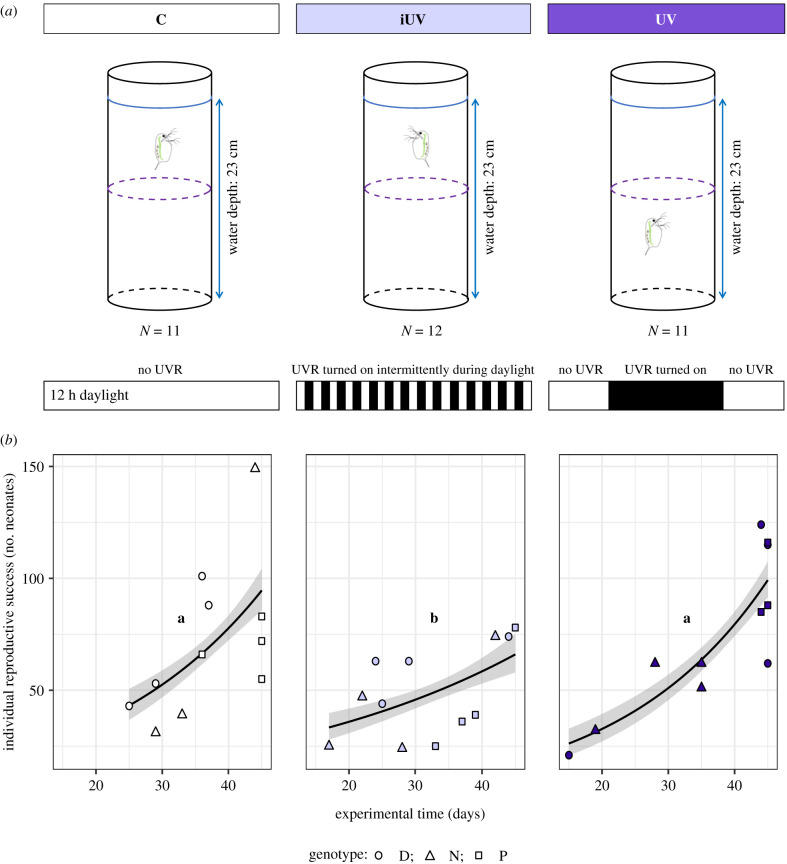
(*a*) Diagram of the experimental design showing the three treatments: control (C), in white, exposed to cool white light and no UVR. Intermittent UVR treatment (iUV), in lilac, exposed to constant cool white light and fluctuating UVR, which was turned on and off every 15 min during daylight. In violet, constant UVR treatment (UV) exposed to cool white light and constant UVR during 6 h during daylight. ‘*N* =’ shows the number of replicates, and the dashed line in the middle of the aquaria represents the criterion for registering *Daphnia* position as ‘bottom’ or ‘surface’ during the behavioural recordings. (*b*) Individual reproductive success (total number of neonates produced per female during the experiment) for each treatment: control (white symbols), intermittent UV (lilac) and constant UV (violet). Different-shaped symbols indicate different genotypes. The black line represents a Poisson curve adjusted to data, and the grey shading, the confidence interval (95%). Different letters (a, b) in the graphs denote significant differences between treatments (GLM Poisson, *χ*^2^(d.f.) = 13.594(2), *p* = 0.001; Tukey's test, *p* < 0.05).

Juvenile female *Daphnia magna* (8 days old) were isolated from laboratory cultures that had been kept under constant light and temperature conditions without UVR. Three *D. magna* genotypes were used in this experiment, originally isolated from different lakes in southern Sweden. Each treatment had all three genotypes represented, with each genotype replicated at least three times per treatment (figure 1*a*). The individuals were isolated from the third brood of single mother per genotype. The experiment was terminated at the point when fewer than three individuals were present in all treatments (45 days).

Individual females were placed into an experimental aquarium (plexiglass tube 25 × 10 cm, height × diameter; water volume = 1635 ml), where they were kept throughout the experiment at a constant temperature of 19 ± 1°C and a 12 L: 12 D photoperiod, and were fed with live *Tetradesmus obliquus* (Chlorophyceae), ad libitum (details in the electronic supplementary material). To ensure standardized environments, the water in all aquaria was replaced once a week with fresh, aerated water and *T. obliquus*.

The treatments were control (C), intermittent UVR (iUV) and constant UVR (UV) (figure 1*a*). UVR was provided using one lamp (UVA-340 nm; *Q*-panel, radiation = 108.1 ± 23.5 µW cm^−2^), and daylight was provided throughout the duration of the photoperiod via a combination of cool white lamps (OSRAM L, 18W/21-840 and AURA T8 36W/830, radiation = 36.2 ± 6.2 µmol m^−2^ s^−1^). All the treatments were exposed to the same daylight intensity over the 12 h light part of the photoperiod, whereas the iUV was exposed to UVR for two periods of 15 min every hour throughout the day, mirroring fluctuating sunlight, and the UV treatment was exposed to constant UVR during 6 h a day (figure 1*a*), resembling a sunny day without cloud cover. The position of the aquaria within each treatment was randomized twice a week.

To determine the effects of the UVR and fluctuating exposure, *Daphnia* survival and reproduction were monitored during the entire experiment. *Daphnia* survival was checked every day and offspring were removed from the aquaria twice a week.

To assess *Daphnia* swimming behaviour, the individual position in each aquarium was registered as ‘bottom’ or ‘surface’ when the animal was below or above a line drawn at the middle of the aquarium (figure 1*a*). The recordings were initiated just before the UV radiation was turned on in the iUV treatment, followed by a recording about 30 s after the UV was turned on. The recordings in all treatments followed this schedule, summing up to 46 behavioural recordings during 11 h in each treatment, on four recording occasions during the experimental period.

### (a) Data analysis

All analyses were performed using R v. 3.5.1 [18], and figures were drawn using the package ‘tidyverse’ [19]. *Daphnia* survival was analysed as a dependent variable, registered as the day of death for each individual. Survival analysis was performed using the package ‘survival’ [20], and Cox proportional hazard regression model, using a survival object (day of death and survival status) as the dependent variable, and treatment and genotype were used as explanatory variables.

The reproductive success of each *Daphnia* was assessed as the total number of neonates produced per female until the end of the experiment (day 45). A generalized linear model (GLM) with Poisson error distribution was used to evaluate the effects of treatments on total *Daphnia* reproduction, including also time and genotype as explanatory variables.

For the behavioural analysis, the dependent variable was the total number of changes in position performed per individual female at each sampling date, and it was analysed using a generalized linear mixed model (GLMM) with Poisson error distribution, using the package ‘lme4’ [21]. Date, treatment and genotype were the explanatory variables, and the individual *Daphnia* was used as random effect. All R packages used during the analysis are detailed in electronic supplementary material, table 1.

## 3. Results

Considering *Daphnia* survival, there were no significant differences among treatments (table 1 and electronic supplementary material, figure S1). However, individuals exposed to the fluctuating UVR treatment (iUV) showed the lowest reproductive success, measured as the total amount of neonates produced during the experiment (table 1, Tukey's test, *p* < 0.05; figure 1*b*). On the other hand, the UV treatment had a similar reproductive output compared with unexposed controls (Tukey's test, *p* = 0.533). The fluctuating UVR exposure reduced *Daphnia* reproductive output overall. *Daphnia* genotype was a significant variable in both survival and reproduction models (table 1).

**Table 1.** Results of Cox proportional hazard model, GLM and GLMM for survival, reproduction and behaviour, respectively. LR *χ*^2^ tests (*χ*^2^), degrees of freedom (d.f.) and *p-*value for each explanatory variable and interactions are shown.

dependent variable	explanatory variable	*χ*^2^	d.f.	*p-*value
survival	treatment	3.216	2	0.200
	genotype	14.321	2	<0.001
reproduction	time	217.566	1	<0.001
	treatment	15.263	2	<0.001
	genotype	46.909	2	<0.001
	time × treatment	13.594	2	0.001
behaviour	date	1.420	1	0.233
	treatment	30.314	2	<0.001
	genotype	23.735	2	<0.001
	date × treatment	3.981	2	0.137

**Figure 2.**
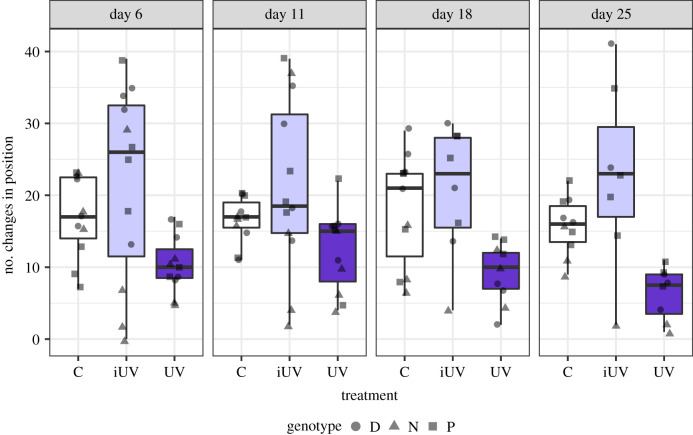
Number of changes in position between treatments for each behaviour recording day. The grey symbols represent the data from each individual *Daphnia* and different-shaped dots indicate different genotypes. The boxplot shows the median for each group as a black horizontal line, the first and third quartile with the box, and the minimum and maximum with the vertical lines.

The individuals exposed to the UV treatment (6 h of continuous dose of UVR) performed the lowest number of changes in position (table 1, Tukey's test, *p* < 0.001; figure 2) and were more often in the lower section of the aquarium compared with the iUV treatment group (electronic supplementary material, figure S2), whereas there was no difference in the number of changes in position in the iUV treatment compared with the controls (Tukey's test, *p* = 0.761; figure 2 and electronic supplementary material, figure S2). Similar to the life-history models, *Daphnia* genotype was a significant variable in the model (table 1). When analysing differences in behaviour throughout the experiment, date as an explanatory variable was not significant, i.e. we found no evidence for behavioural plasticity.

## 4. Discussion

Despite the importance of solar UVR in aquatic ecosystems [5,22], the consequences of natural fluctuations in UVR are still unclear. Threat responses to UVR have been repeatedly demonstrated among small invertebrates, such as *Daphnia* [6,15]. As organisms need to allocate their limited energy to body maintenance/growth, reproduction and movement, any energy diverted to repeated threat responses will not be available for the aforementioned activities [8]. We provide here the first evidence that the variability associated with how an environmental stressor is delivered causes aquatic invertebrates to adopt alternative strategies, leading to population-level consequences.

It is well established that both UV-A and UV-B solar radiation have adverse effects on zooplankton [23]. While UV-B radiation has the potential to damage most biological macromolecules (including DNA [24]), UV-A radiation generates several by-products that cause oxidative stress to numerous cellular components [25]. Owing to the predominant form of UVR in this study being UV-A, it is plausible to assume that the constant production of damaging chemical by-products required equally constant repairing at the molecular level. In zooplankton, this could be achieved through the utilization of either the energetically costly nucleotide excision repair process, or the less costly photo-enzymatic repair pathway, the latter being specifically induced by UV-A radiation [26]. It has been demonstrated that the induction of these systems increases the survival of individuals [27], and this could explain the absence of differences when considering survival between treatments.

The cost of the repair process could instead be covered by the redistribution of energy from other life-history traits, such as reproduction. Our results showed that individuals exposed to fluctuating UVR reduced the number of offspring produced in comparison with both non-exposed individuals and, more interestingly, those that were constantly exposed to UVR. This indicates that the dose of UVR did not determine the reproductive success; instead, it was the temporal variability of the stressor driving the different responses. As environmental variability can influence population growth and fitness in many interrelated ways [28], the fluctuating environment in our experimental set-up could have been difficult for the organisms to predict and respond to accordingly and indeed affected the individual fitness. Our results suggest that despite the presence of the stressor in both UVR-exposure treatments, the constant environment could represent a more benign environment. The predictability of the stressor may allow behavioural adaptations to offset fitness costs.

Behavioural responses are well documented in zooplankton exposed to UVR [5–7]. In contrast to the reproductive output, the behaviour of individuals exposed to fluctuating UVR closely resembled the behaviour of the non-UVR-exposed *Daphnia*. Constantly exposed individuals, however, showed a dramatic reduction in daily movement. We considered the possibility that over time *Daphnia* can plastically adapt behaviourally, but we found no evidence supporting plastic behavioural responses in this experiment. *Daphnia* have long been established to be negatively phototactic, with extreme avoidance of UVR [9]. UVR has, in fact, been proposed as one of the key drivers in the iconic diel vertical migration pattern that *Daphnia* and many other zooplankton perform [6]. Although the costs of diel vertical migration have been assumed negligible [29,30], there has been some controversy over the energetics of such movements [8]. Our results clarify this by showing that the movements to repeatedly avoid UVR may, indeed, increase energy demands. This is based on the observation that the iUV-treated group was the only one that had a reduction in reproductive output. On the other hand, the group exposed to constant UV showed reduced movement, staying in deeper water during exposure, with no identifiable consequence to reproduction.

We recognize that in more natural settings, the heterogeneity of the environment would allow behaviour to play a larger part in determining the optimal strategy for maximizing fitness. For example, diel vertical migration, for which many zooplankton species are renowned, drastically alters exposure to UVR. Hence, extrapolating behavioural results from controlled experiments into natural environments should be done with caution. Despite these precautions, we show here that *Daphnia* have the potential to adopt alternative strategies for dealing with either constant exposure or repeatedly fluctuating UVR, and the response to the more variable environment represents a higher reproductive cost. It has been demonstrated that other threats, such as predation, can cause rapid, local adaptations [31,32]; in a broader context, the significant effect of genotype in our study implies that, depending on how variable a stressor is in nature, the population responses can be different, generating a framework that likely can promote rapid population differentiation and local adaptation.

## Data accessibility

The datasets supporting this article are available in the Dryad Digital Repository: https://dx.doi.org/10.5061/dryad.fttdz08t3 [33].
